# Aspirated Almond Masquerading as an Obstructing Endobronchial Mass Suspicious for Lung Cancer

**DOI:** 10.1155/2018/3742036

**Published:** 2018-06-07

**Authors:** Sreeja Biswas Roy, Mitchell D. Ross, Nikhil Madan, Hesham Abdelrazek, Rebekah Edwards, Earle S. Collum, Ross M. Bremner, Vipul J. Patel, Tanmay S. Panchabhai

**Affiliations:** ^1^Department of Internal Medicine, St. Joseph's Hospital and Medical Center, Phoenix, AZ, USA; ^2^Norton Thoracic Institute, St. Joseph's Hospital and Medical Center, Phoenix, AZ, USA; ^3^Department of Pathology, St. Joseph's Hospital and Medical Center, Phoenix, AZ, USA

## Abstract

Foreign body aspiration is relatively rare in adults compared to children. In adults with delayed presentation, a history of choking is often absent, resulting in delayed diagnosis and significant morbidity. Common presenting features in adults include nonresolving cough with or without fever, hemoptysis, or wheezing and may mimic infectious, inflammatory, or neoplastic disorders. We present a case of a 64-year-old man with 80-pack-year smoking history who had a nonresolving left lower lobe infiltrate on chest radiograph after treatment for community-acquired pneumonia. His insidious-onset symptoms included cough, decreased exercise tolerance, and localized wheezing. Computed tomography of the chest showed a left lower lobe consolidation, with narrowing of the bronchus. Flexible bronchoscopy revealed a fleshy endobronchial mass, prompting endobronchial needle aspiration and biopsies, all of which revealed acute inflammation on rapid onsite evaluation. After multiple biopsies, a white pearly object with a detached brown cover was revealed; the object was found to be an aspirated almond. The almond and its peel were retrieved. The patient acknowledged that he had frequently eaten almonds in the supine position while recovering from a previous injury. His symptoms completely resolved at 3-month follow up, and he has ceased smoking and no longer consumes food while supine.

## 1. Introduction

Foreign body aspiration is rare in adults, with roughly 0.16 to 0.33 cases reported per 1000 individuals [1]. Adult cases contribute to less than 20% of all reported foreign body aspiration events [2], in part because adults often present insidiously, without the telltale prior history of choking [3]. The diagnostic approach for these patients is directed by their clinical presentation, which may be acute or chronic. Diagnosis of foreign body aspiration is often delayed in adults, due to the nonspecific nature of symptoms, nonemergent presentation, and broad range of differential diagnoses (e.g., unresolved infectious pneumonia, lung abscess, obstructive pneumonia secondary to inflammatory disorders, or neoplasms). Risk factors for aspiration may help point to the diagnosis but may not always be evident, thus delaying a diagnostic or therapeutic bronchoscopy. A low threshold for early bronchoscopy may prevent significant morbidity and complications such as recurrent or nonresolving pneumonia, obstructive atelectasis, bronchiectasis, and pneumothorax [4]. Flexible bronchoscopy may be used in adults for diagnosis and retrieval, whereas rigid bronchoscopy is the preferred modality in pediatric patients. We present a rare report of a man with a significant smoking history who had aspirated an almond, presenting with worsening airflow obstruction and a fleshy endobronchial lung mass.

## 2. Case Presentation

A 64-year-old-man with an eighty-pack-year smoking history presented with insidious onset of worsening shortness of breath over a period of 6 months. He had previously undergone ablation for atrial fibrillation, as well as surgery and radiation for prostate cancer. Six months before presenting to our institution, he had fallen and fractured 2 right-sided ribs and developed an upper respiratory tract infection one month later. Over the next 3 months, the patient continued to experience intermittent fevers, chills, cough, wheezing, and exertional dyspnea.

**Figure 1:**
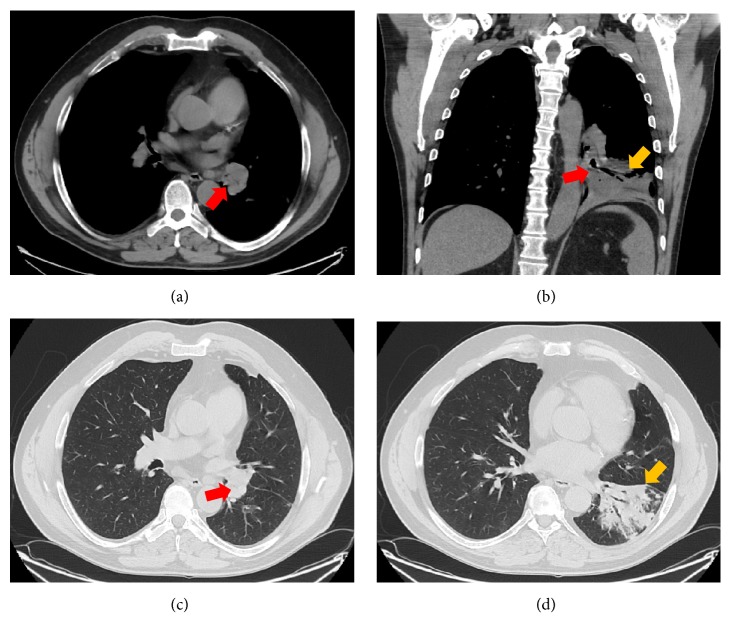
Computed tomogram (CT) of the chest (mediastinal window) in horizontal view (a) and coronal view (b) showing a narrow left lower lobe (LLL) bronchus (red arrow) and distal postobstructive consolidation (yellow arrow). CT of the chest (lung widow) in horizontal view ((c) and (d)) also depicting the narrowed LLL bronchus (red arrow) with the distal consolidation (yellow arrow).

The patient was initially treated with oral antibiotics for presumed community-acquired pneumonia; however, his symptoms persisted despite multiple courses of antibiotics and inhaled bronchodilators, and he was ultimately hospitalized. Serum serology for coccidioidomycosis was negative. Sputum cultures grew normal upper respiratory flora. His chest radiograph showed a left lower lobe (LLL) infiltrate. A computed tomogram (CT) of the chest showed a LLL consolidation, with evidence of narrowing of the LLL bronchus (Figure 1). There was an abrupt cutoff in the LLL bronchus, but no endobronchial lesion was detected (Figure 1). A bronchoscopy performed at the hospital showed a “rounded, nonulcerating, pink mass-like growth” obstructing the LLL bronchus. Brushings from the growth were negative for malignancy, but no biopsies were obtained. He was referred to our center for evaluation of this endobronchial lesion.

Upon further examination, the patient recalled a significant decline in his exercise tolerance about 6 months prior to the current presentation. His pulmonary function tests showed severe airflow obstruction, with FEV_1_ of 1.53 L (42% predicted), which was a decline from his previous FEV_1_ of 2.2 L (63% predicted) 6 months earlier. The patient's physical examination revealed decreased breath sounds at the lower left lung base, with wheezing limited to the LLL. Although he had continued to smoke for the past 6 months, he had quit smoking 3 weeks prior to the current presentation. Based on his symptom complex, worsening airflow obstruction, and the mass seen on prior bronchoscopy, the likely differential diagnoses included primary lung cancer, lung carcinoid tumor, and foreign body aspiration. Although foreign body aspiration was unlikely, the development and progression of his symptoms over just 6 months would also be unusual for primary lung cancer or for lung carcinoid tumor.

**Figure 2:**
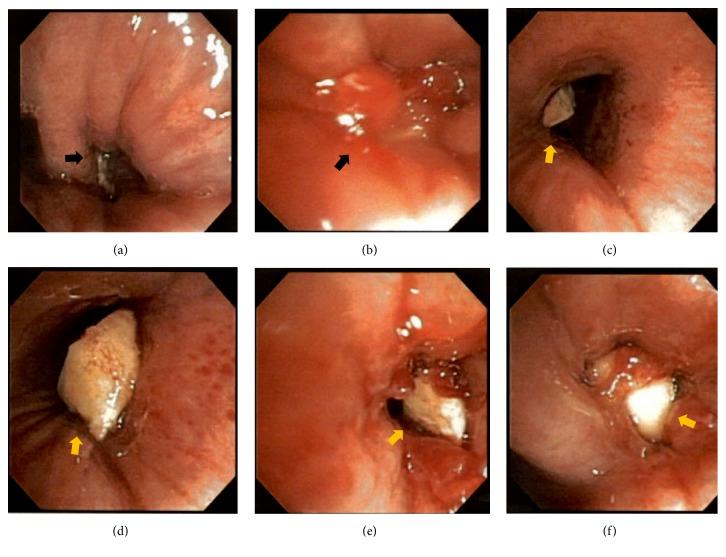
Bronchoscopic images ((a) and (b)) depicting an obstructed LLL bronchus with a fleshy, pearly endobronchial mass (black arrow). Bronchoscopic images of the almond (foreign body) in the left main stem and LLL bronchi [(c)-(f); yellow arrows].

**Figure 3:**
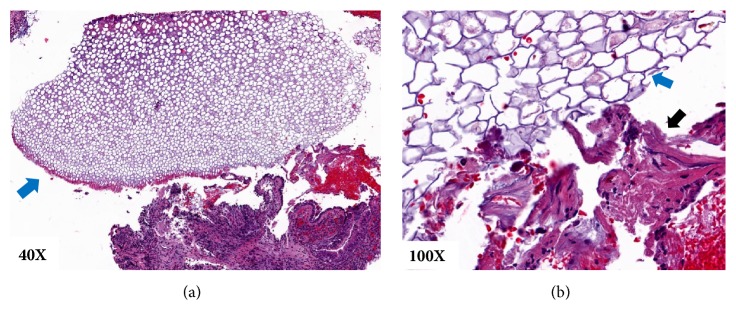
Hematoxylin and eosin stained images of the foreign body show vegetable matter with thick cell walls associated with plants (blue arrow) and acute inflammation with tissue debris (black arrow) under 40x (a) and 100x (b) magnifications.

The patient underwent another bronchoscopy, which revealed a narrow LLL bronchus, with a fleshy endobronchial mass lesion (Figure 2). Multiple fine needle aspirates were obtained using a Wang needle (CONMED, Utica, NY), but every pass was positive for dense acute inflammation on rapid onsite evaluation. Multiple endobronchial biopsies were then obtained, and 2 pearly white structures with a brown detached cover were revealed (Figure 2). The object was extracted in its entirety using a Zero Tip Airway Retrieval Basket (Boston Scientific, Marlborough, MA) and biopsy forceps; the object was identified as an aspirated almond. An ultrathin bronchoscope was then used to examine the airways distal to the aspirated almond, and there was no evidence of any additional foreign material. A bronchoalveolar lavage was performed at the end of the case, and specimens were sent for culture. Pathology of the extracted foreign body was consistent with vegetable matter (Figure 3), and bronchoalveolar lavage cultures were negative for bacterial or fungal organisms.

**Figure 4:**
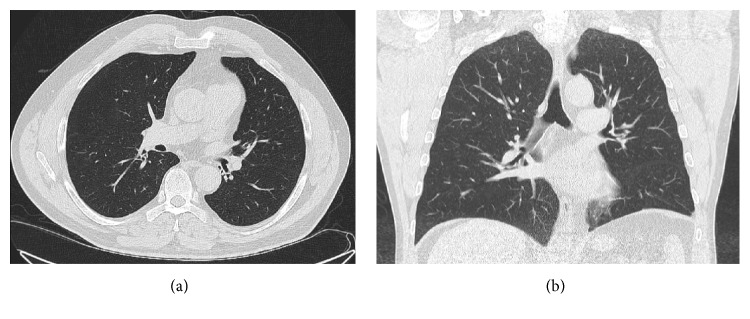
CT scan of the chest (lung window) in horizontal (a) and coronal (b) sections showing a normal lung parenchyma with complete resolution of the left lower lobe infiltrate noted in Figure 1.

A 10-day course of amoxicillin-clavulanate was prescribed due to the imaging evidence of aspiration pneumonia secondary to almond aspiration. On follow-up examination one month later, he reported drastically increased exercise tolerance and his cough and wheezing had disappeared; meanwhile, pulmonary function tests showed an increase in his FEV_1_ to 2.12 L (60% predicted), and a repeat flexible bronchoscopy revealed no bronchial stricture. The patient shared that while he was recovering from his rib fractures, his favorite snack (enjoyed in the supine position) was almonds. While foreign body aspiration for 6 months could lead to irreversible parenchymal changes, a follow-up CT scan at 3 months revealed complete resolution of parenchymal infiltrates in this case (Figure 4). At the time of his 6-month follow-up, his exercise tolerance had returned to baseline and he had successfully quit smoking.

## 3. Discussion

The presentation of patients with foreign body aspiration differs between adults and children. Although foreign body aspiration in children is more commonly symptomatic and often presents as asphyxiation after a choking episode, presentation in adults is often subtle and chronic. The classic “penetration syndrome” described in children is absent in adults, and more than 50% often cannot recall the aspiration event, which often delays the diagnosis [5]. Risk factors for foreign body aspiration in adults include older age, medications, or neurological conditions that would impair cough and swallowing and loss of consciousness secondary to alcohol intoxication or trauma [1, 3]. Common presenting symptoms include an unresolved cough (with or without fever), hemoptysis, chest pain, wheezing, or dyspnea [3]. The most commonly reported chest radiographic findings include nonresolving consolidation or segmental collapse; a foreign body is visualized in only a quarter of cases [1].

A chest CT is often helpful in identifying the foreign body, which in turn guides procedural planning. Flexible bronchoscopy is the logical next step, and this can be diagnostic as well as therapeutic. Retrieval techniques include flexible or rigid bronchoscopy, but great care must be taken to prevent pushing the foreign body distally into the airway [3]. In general, flexible bronchoscopy is preferred in adults, as it has greater than 90% success rate of retrieval and allows for a comprehensive airway examination [3, 6, 7].

The present case depicts a rare scenario in which the patient had no recollection of the aspiration event and did not relay history that ultimately would have been helpful in making the diagnosis. Foreign body aspiration can lead to postobstructive pneumonia due to airway obstruction or due to vegetable matter causing aspiration syndromes. In the present case, the postobstructive pneumonia due to LLL obstruction led to the patient seeking medical attention, which ultimately led to the discovery of the aspirated almond. More importantly, the essential oils found in nuts such as almonds are irritants that result in dense inflammatory changes, which is probably what caused the fleshy appearance of the mass. Although the endobronchial mass observed on bronchoscopy was initially concerning due to the patient's 80-pack-year smoking history, its rapid growth was not characteristic of primary lung cancer or a lung carcinoid tumor. Still, because critical information was left out in the patient's history, diagnosis of the foreign body aspiration—a rare diagnosis in the first place—was not intuitive.

The present case has the following learning points: (1) foreign body aspiration in adults can have varied presentations, and the aspiration event is often not memorable for patients; (2) flexible bronchoscopy is the diagnostic and therapeutic modality of choice for adult patients with foreign body aspiration, and (3) rapidly worsening airflow obstruction should point toward endobronchial airway obstruction. Delayed diagnosis of foreign body aspiration can lead to severe complications, including empyema, lung abscess, pneumothorax, airway obstruction, hemoptysis, and pneumothorax [2, 3].
